# Thriving from Work Questionnaire: Validation of a Measure of Worker Wellbeing Among Older U.S. Workers

**DOI:** 10.3390/ijerph22091428

**Published:** 2025-09-12

**Authors:** Maren Wright Voss, Cal J. Halvorsen, Kanchan Yadav, Stephanie M. Neidlinger, Gregory R. Wagner, Susan E. Peters

**Affiliations:** 1 Department of Social and Behavioral Sciences, Harvard T.H. Chan School of Public Health, Harvard University, Boston, MA 02115, USA; mvoss@g.harvard.edu (M.W.V.); kanchan_yadav@hsph.harvard.edu (K.Y.); 2 Center for Work, Health, and Wellbeing, Harvard T.H. Chan School of Public Health, Harvard University, Boston, MA 02115, USA; chalvorsen@wustl.edu; 3 Brown School, Washington University in St. Louis, St. Louis, MO 63130, USA; 4 Unit of Occupational Medicine, Institute for Environmental Medicine, Karolinska Institute, 17177 Stockholm, Sweden; 5 Department of Work, Organizational, and Business Psychology, Helmut-Schmidt University, 22043 Hamburg, Germany; neidlinger@hsu-hh.de; 6 Department of Environmental Health, Harvard T.H. Chan School of Public Health, Harvard University, Boston, MA 02115, USA; gwagner@hsph.harvard.edu

**Keywords:** work-related wellbeing, psychological safety, item response theory, psychometrics, work-life balance, occupational stress, job satisfaction, working conditions, psychological well-being, occupational health

## Abstract

As life expectancy and retirement ages rise globally, understanding how older workers thrive in the workplace is an increasingly vital measurement and wellbeing priority. In this study, we validated the Thriving from Work Questionnaire (TfWQ) for workers aged ≥50. A U.S. online panel yielded 617 older workers and 372 younger counterparts for comparison. Using item response theory alongside model-fit evaluation and correlational tests with job/life satisfaction, engagement, burnout, and turnover intent—we assessed reliability and construct validity of the long- (30 reduced to 29-item) and short- (8-item) form TfWQ versions. We recommend omitting one of the original items from the long-form for use in older workers. Instrument reliability was high (α = 0.94 long-form; 0.90 short-form). Model fit was established for both long- and short-form versions with acceptable model fit indices. Convergent validity was supported by strong, theory-consistent correlations with the external constructs. Older workers, compared with those 20–49 years, had higher scores of thriving from work as well as differences identified on nine items. These age-patterned differences highlight actionable levers for occupational-health age-sensitive policy, wellbeing interventions, and workforce planning. The TfWQ offers a robust, reliable, valid, and practically oriented tool for evaluating older workers’ wellbeing with utility across research, practice, and policy.

## 1. Introduction

Global gains in longevity are extending average working life years and pushing retirement to later ages. Labor force participation among older workers has risen continuously in the US since the 1980s [1], when federal policy lifted most earnings penalties for working past Social Security full-benefit age [2]. Similar trends are visible elsewhere: 19 of 34 OECD countries have legislated higher pension-eligibility ages in the past two decades [3], with further statutory age increases expected [4]. Worker wellbeing is a construct that addresses the conditions that allow workers to thrive and optimize wellbeing in the workplace [5,6,7]. Work and aging are not antithetical [8,9,10], yet age-specific barriers must be addressed if societal goals for extended careers are to be fully realized. Assessing the work-related factors and conditions to support an aging workforce calls for a work-related wellbeing measure that broadly conceptualizes and measures wellbeing [11,12].

The Thriving from Work Questionnaire (TfWQ) is a wholistic measure of work-related wellbeing that focuses on the positive contributions of work toward overall thriving in life [13]. This questionnaire differs from other instruments in that it does not focus explicitly on poor wellbeing (e.g., burnout, or poor mental health), nor is it uni-dimensional (e.g., job satisfaction, exhaustion, or work engagement). The TfWQ has been validated as a measurement tool of worker thriving for the general working age population across multiple industries and countries [13,14,15,16], but has not been evaluated specifically for relevance to characteristics of older workers, whose experiences and approaches to work may differ due to their life-course-specific socioeconomic and health circumstances. If valid in an older worker population, the TfWQ has implications for use informing countries and organizations considering the health impact of longer work policy, evaluation of interventions focused on older workers, and to better understand the conditions influencing older workers’ thriving.

### 1.1. Measurement Considerations in Aging

Using assessments to measure concepts like worker well-being in older workers requires attention to age-salient response processes and sampling. At the respondent level, aging may influence comprehension and judgment in psychosocial measures: cognitive slowing, wider health heterogeneity, lower average educational attainment, limited internet access, and stronger social-desirability concerns may introduce distinctive response and sampling biases [17]. Socio-emotional selectivity theory posits a foreshortened time horizon that shifts goals and emotional valence, altering how items about growth, meaning, and future-orientation might be interpreted [18,19,20]. Age-related shifts in functional capacity (e.g., sensory changes, muscular strength, immune function, memory/processing speed) co-occur with developmental gains in emotion regulation and conscientiousness; fluid abilities (e.g., reasoning and adaptability) often decline while crystallized knowledge and skills continue to develop over one’s life [21,22]. Systematic age gradients in worker attitudes and behaviors underscore the need for age-sensitive measurement strategies [23,24,25]. Healthy-worker survivor processes can further bias samples toward individuals who remain employed despite health limitations [26].

Beyond individual changes, labor-market structures shape whether older adults thrive from their work [27]. Labor-force participation falls after midlife due to voluntary exits and fewer viable job openings [1,28,29,30,31]. For those who remain, work can often be more precarious or contingent [32,33], and ageism—both subtle and explicit—might restrict access to good jobs [34]. Early retirement may be driven by disability, job loss, or peak caregiving responsibilities in the “sandwich generation” [31,35,36,37,38]. These structural and individual dynamics together motivate the need for instruments that (a) use clear, low-burden wording and cognitive processing, (b) are attuned to late-career priorities (e.g., meaning, knowledge transfer), and (c) capture modifiable conditions of work such as autonomy, schedule control, and person-environment fit that have been considered important to older workers.

### 1.2. Development of the Thriving from Work Questionnaire (TfWQ)

Building on the age-salient considerations outlined above, the TfWQ was originally developed to capture the conditions and experiences relevant to and with broad application across industries, jobs, and worker demographic factors such as age [13]. Thriving from work (TfW) is defined as a state of positive mental, physical, and social functioning in which workers’ experiences of their work and working conditions enable them to thrive in their overall lives, contributing to the ability to achieve a full potential in work, home, and community [13]. It is conceptualized across six domains of work-related wellbeing (see Table 1). To date, for the U.S. worker English-speaking population, there is a short-form questionnaire containing 8 items that measures the latent thriving from work construct to generate a TfW general score. A long-form questionnaire contains 30 items across six domains. The domains can be used to interpret the contexts of thriving and the highest priority areas to enhance wellbeing.

The TfWQ was developed using rigorous and iterative methods to define and conceptualize the underlying model that supported the development of the original questionnaire [13,14]. Development and validation of the Thriving from Work Questionnaire in general worker populations and the systematic and rigorous survey methods used have been described in prior publications [13,14,39,40]. Earlier validation studies have focused on different geographical contexts and languages, including Germany [16] and Spanish-speaking Latin American countries [15]. The TfWQ has also been validated in sector specific worker samples including in a large micro-finance worker sample [15] as well as healthcare, public health workforce, and fulfillment centers. To date, the questionnaire has not been validated specifically in older workers who, as described in Section 1.1, have age-salient characteristics that may influence their responses and understanding of the items.

### 1.3. Thriving from Work Questionnaire (TfWQ) Validation in Older Workers

Recognizing the presence of age-dependent individual and structural measurement invariances, validating a wellbeing instrument specifically in an older worker population can address: (a) life-stage differences (e.g., approaching retirement, caregiving, etc.), (b) changes in health status or function, and (c) socio-cultural factors (e.g., generational values, time-related expectations, etc.). This validation helps ensure reliability and construct relevance given population-specific tendencies that alter interpretation or item perceptions. Accurate assessment can help organizations develop targeted interventions to improve wellbeing among older workers, supporting their health, engagement, and retention in the workforce. Applying an aging workforce lens to Thriving from Work is an opportunity to evaluate legitimate concerns for aging workers (e.g., [41]) against wellbeing from work literature to explicitly assess implications for older worker thriving [42,43]. Use of a comprehensive work-related wellbeing assessment validated for older workers may offer insights for policy to protect older workers being pushed toward unwanted longer work lives or premature exit from the workforce.

The purpose of this study is to validate and evaluate the psychometric properties of the long-form and short-form versions of the TfWQ in a working population aged 50 years and older, and to compare these with the questionnaires’ properties when used with younger workers. We focus on workers 50 years and older as an oft-used minimum age for researching older workers [44]. We address two main research questions relevant to aging worker applications of the TfWQ:

1. Does the Thriving-from-Work Questionnaire (long-form and short form) retain acceptable reliability and factorial validity when administered to workers aged 50 years and older?
2. Do workers aged 50 years and older systematically report differences in domains or aspects of thriving from work compared to their younger working counterparts?

## 2. Materials and Methods

### 2.1. Methodological Approach

We validated the TfWQ for older workers following the validation approach used in previous TfWQ validation studies [14,15,16]. We employed an Item Response Theory (IRT) bi-factor model, assessing local dependence of items and the associated residuals for each factor [45,46,47]. The TfW general factor is the latent trait to be measured and is represented in the common variance across TfWQ items. IRT was selected for two reasons. First, empirical evidence from meta-analysis supports the superior fit of using IRT for bifactor models relative to confirmatory factor analysis (CFA), exploratory structural equation modeling (ESEM), and hierarchical variants, particularly when considering theoretical coherence and empirical fit [48]. Second, prior validation studies of the TfWQ used IRT bi-factor model methods, which allow us to directly compare our findings with prior validation results. Of note, the IRT bi-factor method is considered similar to confirmatory factor analysis [49], in that relative weights of each item can be calculated for the general factor and for each TfW domain factor. The domains are represented by the unique variance associated with the specific factors, which may be related to a dimension of the latent trait [46]. Although IRT model fit statistics are used and reported within the manuscript, Appendix B (Table A2 and Table A3) provides comparable factor loadings for those who are more familiar with the CFA statistical approach.

IRT requires sufficient responses per item to ensure stable and reliable estimation of item parameters across the latent trait continuum [50]. To address this, we used an age-stratified sampling approach mirroring the original validation design. Specifically, we planned for 300 respondents aged 50–59 and 300 respondents aged 60 years and older, yielding at least 10 responses per item per subgroup for the 30-item TfWQ survey. This stratification and sample size ensured adequate power for IRT modeling and allowed for stable parameter estimation with meaningful comparisons across older age groups.

We assessed construct validity, hypothesizing expected performance on similar and dissimilar construct measures by degree of relatedness to TfWQ [51]. We measured convergent validity by administering a Cantril self-anchoring ladder as a measure of life satisfaction [52], and a variation adapted for work satisfaction as the ‘Best Job Ladder’. Additionally, we administered a job-related satisfaction measure through a single-item from the Copenhagen Psychosocial Questionnaire [53], work engagement using the Well-BQ 3-item job engagement scale [54], general wellbeing using the Flourishing Scale [12], as well as burnout [55,56,57], and turnover intention. We anticipated negative correlations with burnout and turnover intention, and positive correlations with the measures for life satisfaction, best job, work engagement, job satisfaction, and general wellbeing. Measures are described in Section 2.4. We anticipated modest correlations indicating a degree of similarity, but not so similar as to be highly correlated.

### 2.2. Data Collection

For this study, each participant completed a one-time electronic survey. To recruit for the study and deploy the survey, we used Prolific, an online survey platform specializing in web-based surveys for research in the social sciences [58,59,60]; the survey was open for completion between February and March 2025. The platform allows selection for a nationally representative sample of the U.S. population by age, sex, and ethnicity with active participation in the labor force [61]. Survey compensation for voluntary completion was set to USD$16.

To protect data integrity, we implemented a multi-pronged response-quality protocol targeting insufficient-effort responding. To reduce insufficient effort responding, the use of recommended benign effort messaging was included to remind participants to pay attention and answer honestly [62]. Attention check items directed participants to give a required response, and respondents were notified of attention checking and minimum completion requirements in the survey instructions. Participants were excluded if incorrect responses were provided on two of three interspersed attention check items or if responding to less than 80% of survey items overall. Survey response quality was also enhanced by integrating survey questionnaire design best practices as recommended through the Qualtrics survey platform, which hosted the survey [63]. To ensure each survey responder was a unique participant, multiple responses from the same participants were mitigated by exclusion for repeat geographical location parameters and IP addresses. The survey design employed bot detection to identify participant responses that like a bot, flagged responses for straight lining, and included the use of Google’s RecapchaScore. Prolific’s systematic vetting and registry maintenance process, including the use of ID verification, also helped reduce fraudulent and bot-based responding [64].

### 2.3. Sample Selection

Drawing from the Prolific registry of 104,637 U.S. residents, the sample was delimited by selecting a working population (full-time, part-time, or starting a job within 30 days). An oversampling strategy targeted an additional 25 under-represented participants for every 100 general participants to reflect double representation in four simplified race categories (African American, Asian, multiracial, Native American/Pacific Islander) based upon standard population ratios per the U.S. Census Bureau 2024 population estimates [65]. Sampling was divided into three age groups, with a focus on older workers separated as 50–59 years (300), and age 60+ (300), as well as a younger worker comparison group (20–49 years; 300); we targeted oversampling an additional 75 respondents in each of the three age groups in the minority race and ethnicity categories. This resulted in a request for Prolific to recruit a total of 375 per age group.

The Prolific registry sample pool was sufficient to achieve a representative sample with oversampling for the ‘20–49 years’ working group. For the ‘50–59 years’ group, a representative sample with oversampling was attained for all categories, with the exception of oversampling of Asian respondents. In the ‘60+ years’ group, there was an insufficient Prolific sample registry using the specified age and working status inclusion criteria; resulting in a failure to achieve a representative sample for Hispanic (6.4% attained vs. 19% representative), Asian (1.9% attained vs. 6.6% representative), and Native American/Pacific Islander (1.5% attained vs. 2.5% representative) categories. Additional participants identifying as undefined multiracial were recruited to increase the number of respondents from underrepresented groups. From our target sampling approach, we were able to recruit 375 aged 20–49 years, 352 from 50–59 years, and 265 for the 60+ group.

### 2.4. Validation Measures

All validation measures were measured concurrently on the survey. In addition to the TfWQ and validation constructs, we also asked demographic (e.g., age, race/ethnicity, education) and occupational questions (e.g., occupation, industry/sector, job role, tenure, number of jobs) to allow us to describe the sample (see Section 2.4.8 Demographic and Occupational Variables).

#### 2.4.1. Thriving from Work Questionnaire (TfWQ)

The long-form TfWQ consists of 30 items across six domains that contribute to the Thriving from Work construct (see Table 1). Respondents were asked to rate each TfWQ item on a 6-point Likert scale, ranging from “Never” (1) to “Always” (6), with the option to respond, “Not Applicable” (0). The TfWQ short-form includes a subset of 8 items from the long-form version. During the original TfW validation study and bifactor model analysis, the TfWQ items showing the highest values of marginal discrimination on the general TfW factor, as well as strong theoretical underpinnings from each domain, were selected for inclusion in the short-form questionnaire [13]. Peters and colleagues recommend the use of model-based scoring in research contexts, whereas summative scoring may be more feasible for applied practice settings [66,67]. For descriptive statistics, we used the summed scoring approach for the long-form questionnaire, resulting in a total score of 30–180 for the long form and 8-40 for the short form, with a higher score indicating higher thriving from work.

#### 2.4.2. Life Satisfaction and Best Job

To assess whether one is thriving in their life (or not), we asked participants questions from Gallup’s adapted version of a Cantril self-anchoring ladder numbered from 0 to 10, where 0 represents the worst possible life and 10 represents the best possible life [52]. Participants were also asked to compare their current job with the perceived ‘best job possible’ as per Peters et al. [14]. Participants were asked to indicate which step on the ladder represents their feelings about life in current time, relative to where they think they will stand about five years from now.

#### 2.4.3. Job Satisfaction

To assess satisfaction or dissatisfaction in the current job, we used two single items. We first used an adapted Cantril Ladder, asking participants to indicate which step on the ladder represents their current job relative to their perceived best job, where 0 represents the worst possible job and 10 represents the best possible job. We additionally included a single item from the Copenhagen Psychosocial Questionnaire (COPSOQ-III) [53]. The COPSOQ item asks: “Regarding your work in general, how pleased are you with your job as a whole, everything taken into consideration?” with ranking on a 5-point Likert scale ranging from ‘Very unsatisfied’ (1) to ‘Very satisfied’ (5).

#### 2.4.4. Work Engagement

Participants’ work engagement was assessed with three items from the National Institute of Occupational Safety and Health (NIOSH) Well-BQ [54]. The items were “My work inspires me”, “I am immersed in my work”, and “When I get up in the morning, I feel like going to work”. Items were ranked on a 7-point Likert scale ranging from ‘Never’ (1) to ‘Always’ (7). Cronbach’s alpha for the scale was 0.86.

#### 2.4.5. Overall Wellbeing

To measure overall wellbeing, we used the Flourishing scale, which includes six two-item subscales of Happiness & Life Satisfaction, Mental & Physical Health, Meaning & Purpose, Character & Virtue, Close Social Relationships, and Financial & Material Stability with 12 items in all [12]. Scale administration does not require the last subscale to be administered, which was accordingly excluded to minimize respondent burden. Participant overall flourishing was assessed with ten items from the first five subscales. An example item is, “I understand my purpose in life.” Each item is answered on a 10-point ordinal scale, generally ranging from ‘Strongly disagree’ (0) to ‘Strongly agree’ (10), where answer choices vary depending on item wording. Cronbach’s alpha for the full scale was 0.93.

#### 2.4.6. Burnout

Burnout was assessed with a single item [55,56,57]: ‘Overall, how would you rate your level of burnout?’. The question’s responses included: (1) “Occasionally I’ve been under stress and I don’t always have as much energy, but I haven’t felt burned out”, (2) “I was definitely burning out and have had one or more symptoms of burnout, such as physical and emotional exhaustion”, (3) “I’ve enjoyed my work, with no symptoms of burnout”, (4) “I have felt completely burned out and often wondered if I could go on” and (5) “The symptoms of burnout that I experienced wouldn’t go away. I have been frustrated at work a lot”. Respondents selected the single, best-fitting answer.

#### 2.4.7. Turnover Intention

Participants’ intent to quit was assessed using a three-item scale [68]. The items were: “I would prefer another job to the one I have now,” “If I have my way, I won’t be working for this company a year from now,” and “I have seriously thought about leaving this company.” Items were rated on the 7-point Likert scale from ‘Strongly disagree’ (1) to ‘Strongly agree’ (7). Cronbach’s alpha for the three items was 0.92.

#### 2.4.8. Demographic and Occupational Variables

Whenever possible, we used U.S. Bureau of Labor Statistics (BLS) standard questions for demographic and occupational variables [69]. Demographic information was collected on the survey, including age, race/ethnicity, educational level, and annual household income. For education, we collapsed high school equivalent (e.g., GED) with high school completion. We also collected information on jobs, number of employers, hours worked, occupation, and work role. Work role was collapsed into five categories—Employee (non-manager)/Individual contributor, People manager, Senior leadership (VP/Director), and Executive (C-suite/President), Owner (Owner/Partner); ‘None of the above’ was coded as missing. For job sector grouping, we classified respondents by their occupation rather than their employer industry (e.g., a nurse who works in a factory was classified by their occupation as a nurse). This was because TfWQ domains reflect job- and task-level conditions performed by a specific job role and occupation. Respondents were able to select one of 23 standard occupational classifications (SOC) used by the BLS. This was also cross-checked with their stated occupation, which was provided using an open-text field. The 23 SOC were collapsed into 5-headline occupational categories using the same method employed by the BLS [70], (see Table 2): (i) Management, Professional and Related (e.g., executive, business/finance, technology/math, architecture and engineering, science, legal, education, arts/media, healthcare practitioners), (ii) Sales and Office (e.g., sales, retail, office/administration support, customer service), (iii) Service Occupations (e.g., health-care support, protective service and military, food preparation and serving, cleaning, building and grounds, personal care), (iv) Production, Transportation and Material Moving (e.g., manufacturing and good production, plant operation, logistics, transportation), and Natural Resources, Construction and Maintenance (e.g., farming, forestry, construction, mining, installation/repair).

#### 2.4.9. Statistical Analysis

We assessed the psychometric properties of the TfWQ in an older worker sample by first analyzing item response variability, item difficulty, and variability among respondents. Items with restricted variability or extreme endorsement frequencies were flagged for further consideration. We computed intercorrelations between items to assess the internal structure of the items [71]. TfWQ scale reliability was estimated using empirical reliability coefficients, both for the general TfW factor and for each of the six specific domains.

For the long-form version, we used IRT models using a logistic approximation to estimate marginal discrimination parameters [47]; while for the short version, we specified a unidimensional graded response IRT model, constraining all items to load on the single generalized factor (TfW). Using logistic approximation, we calculated marginal discrimination parameters and a unidimensional graded response IRT model to assess items’ loading on the general factor. Items with high discrimination parameters reflect the ability of the instrument to detect differences in the latent construct, with a threshold of <0.3 as indicating low discrimination and less sensitivity to differences in the latent construct [47]. There were no missing data for Thriving from Work items in the survey after those failing quality checks, as described in Section 2.2, were removed from the dataset. However, ‘Not applicable’ responses on the TfWQ items were treated as missing for analysis. Items were deleted pairwise for other missing data.

We examined the fit of our hypothesized model to the data using common criteria, limited information chi-square statistics (M2 for the long-form/C2 for the unidimensional short-form) for ordinal data, and three widely used structural equation model approximate-fit indices, the root mean squared error of approximation (RMSEA), Standardized Root Mean-Square Residual (SRMR), and the Comparative fit Index (CFI) with recommended cut-off guidelines [72,73,74,75,76]. The selection of a bi-factor model, comprising one general and six specific factors, was guided by theoretical foundations. Competing models were not tested, as the bifactor structure was pre-specified based on prior conceptual work. Cut-off values for the model-fit metrics are not absolute and are interpreted relative to the theoretical background and practical goals of the instrument [77,78]. We selected conventional criteria indicating basic good fit for RMSEA values of <0.08 and CFI > 0.95 [73,77,78]. All analyses were conducted using the ‘mirt’ package in RStudio (Version 2024.09.1+394; Posit Software, PBC, Boston, MA, USA) [79].

## 3. Results

### 3.1. Sample Characteristics

Demographic and occupational characteristics are reported for age-stratified samples: 20–49 years, 50–59 years, and 60+ years (see Table 2). In the table, we stratified these samples into two older worker groups to explore whether workers 60 years and older have different characteristics as they move closer to or beyond U.S. benefits-eligible retirement age.

Older worker sample (n = 617): Older worker participants reported a mean age of M = 58.40 years (SD = 6.46) for those aged 50 and over. 41.0% of the sample were male, 58.0% female, and less than 1% were unreported. Participant ethnicity/race reflecting targeted oversampling included 54.3% White, 4.7% Asian, 21% Black/African American, 11% Latino/Hispanic, 2.3% Native American/Alaskan Native, and 5.8% reporting multiple races. Participants represented diverse work roles such as C-Suite Executive or President (4.54%); Director/Vice President (6.8%); Manager/Senior Manager (33.71%); Individual Contributor (48.46%); and Owner/Partner (6.48%). Most worked in Management, Professional and Related roles (58.67%), followed by Sales and Office (21.6%), Service Occupations (11.7%), Production, Transportation and Material Moving (4.86%), Natural Resources, Construction and Maintenance (2.26%).

Comparison with younger worker sample (n = 372): A comparison sample of working adults aged 20–49 years was recruited for a survey-date-matched comparison group of other-age workers using the Prolific panel registry. Notable differences in the demographic breakdown between older and younger workers were seen in level of education, with higher education levels of undergraduate qualifications and above noted in the younger worker sample. Additionally, the 50–59 year-old workers had higher representation in the over $150,000 annual household income category (18% compared to <10% in 60+ and younger worker samples). Workers over age 60 had a higher representation in the less than $20,000 annual income (12% compared to 7–8% of younger workers). Younger workers had more jobs in the ‘management, professional, and related’ as well as the ‘production, transportation, and material moving’ sectors than the older worker groups. Older workers had more jobs in the ‘service’ and ‘sales and office occupations’. More older workers had ‘owner’, ‘partner’, ‘C-suite/executive’, or ‘Vice President (VP)’, ‘Director’, ‘Manager’, or ‘Senior Manager’ roles. Whereas younger workers were more likely to be in ‘individual contributor’/employee roles. A larger proportion of workers age 60+ worked less than 31 h per week (23% for ages 20–49; 20% for ages 50–59; 38% for ages 60+).

### 3.2. Data Description and Integrity

Less than 1% (n = 3) of Prolific survey completers failed two or more attention check markers, necessitating exclusion from the study, and were removed from the dataset prior to analysis. Three TfWQ items had more than 5% of the sample respond ‘not applicable.’ These items included leave availability and promotion opportunity (Basic Needs subdomain) and commuting (Work-Life Integration subdomain). Non-endorsers of these items differed little in demographic characteristics from the general sample, with small increases in not-applicable responding by those with extreme low income (under $20,000) for ‘promotion’ and ‘leave’ items, and slightly higher endorsement of the ‘safe when commuting’ item by those with high income ($150,000+). The distributions and factor loadings of the leave and commute items were within acceptable ranges. The promotion item showed more responses concentrated at lower values, indicating dissatisfaction and a low specific factor loading (0.28). However, its loading on the general factor was higher than on the specific factor (0.61), suggesting value in retaining ‘promotion’ within the TfW construct measurement for understanding older worker thriving despite some respondents finding it ‘not applicable’ to their own situation. Thus, these items (‘promotion’, ‘leave’, and ‘commuting’) were retained.

Descriptive statistics for TfWQ items were calculated for all items based on a 6-point Likert scale (ranging from Never to Always), with an added ‘Not Applicable’ response that was coded as missing in the analysis. Item distributions for both the TfWQ long-form and short-form items showed a general tendency toward a right skew (higher scores dominant) but were within acceptable parameters (see Table 3). Item skewness values ranged from −1.98 to −0.19, and kurtosis values ranged from −1.01 to 4.39, generally supporting the assumption of approximate normality (see Table 4).

Table 4 describes the descriptive statistics, means, and standard deviations for each of the items by worker age groups (Age 50+ versus Age 20–49), differences between the age groups (see also Section 3.6), as well as the skewness and kurtosis for each item. Item mean averages ranged from 3.15 to 5.32, and standard deviations exceeded 0.90, indicating acceptable response variability. 

In summary, polychoric item intercorrelations (see Appendix A, Table A1) and descriptive statistics (see Table 3 and Table 4) indicate values that were within acceptable parameters.

### 3.3. 30-Item (Long-Form) Version Validation—Item Reduction, Model Validation, and Model Fit

Long-form Item Reduction: The item ‘I worry that I will get hurt at work’ demonstrated little differentiation at the individual trait level (floor effect with low worry reporting). Overall, Polychoric item intercorrelations ranged from 0.17 to 0.92, except for the ‘hurt’ item, which was low, ranging from 0.06 to 0.60, with an average item intercorrelation of 0.21 (see Appendix A, AI). Additionally, the ‘hurt’ item had a low marginal discrimination parameter for the general factor of TfW (0.44). Removing the ‘hurt’ item based on its poor performance in the older working sample improved model fit substantially as expected. All other item intercorrelations were close to or within the acceptable range (0.30 to 0.80), suggesting moderate to strong associations among items without excessive redundancy (>0.90) (see Appendix A, Table A1). This resulted in a recommended long-form instrument of 29 (of the original 30) items for use in older workers, with the omission of the ‘hurt’ item (item 29).

Reliability: The long-form version of the TfWQ demonstrated high empirical reliability (0.94) in the older worker (50+) sample, indicating strong internal consistency and robust measurement of the shared variance across the retained 29 items. Three of the specific factors additionally showed moderate to strong reliability, ranging from 0.65 to 0.84 (Emotional and Psychological Wellbeing, Work-life Integration, and Job Design), indicative of capturing the unique variance for each dimension. These three domains potentially also could be considered as standalone measures of these dimensions (see Appendix B, Table A2). Three specific factors (Social Wellbeing, Basic Needs, and Health, Physical, and Mental Wellbeing) exhibited lower reliability scores (0.50–0.58). As the instrument is designed to capture the general TfW construct, the high general factor reliability suggests the instrument is well-calibrated and robust in achieving the primary measurement objective. Thus, the long-form version of the TfWQ was found to be a reliable measure of older worker wellbeing.

Bi-Factor Model Validation: The marginal discrimination parameters for items loading on the general TfW factor were acceptable, ranging from moderate (commute, 0.94) to high (fair treatment, 3.63) (see Table 5). Marginal discrimination parameters for items loading on each of the domains largely fell within an acceptable range (<0.3 to 1.0) for items in five of the domains (1.01 to 1.83 for Emotional and Psychological Wellbeing; 0.39 to 0.99 for Social Wellbeing from Work; 0.63 to 1.96 for Work Life Integration; 0.34 to 0.96 for Basic Needs and Thriving; and 0.37 to 1.14 for Health, Physical, and Mental Wellbeing from Work). For the Job Design & Experience of Work domain, all items were between 0.35–1.28, with one item being below 0.3 (resources, 0.11). Loadings of IRT discrimination parameters are available in Appendix B (see Table A2). All marginal discrimination parameters for items loading on the general factor were acceptable; only one item loading onto the Job Design domain was classified as low, which may warrant further examination.

Model Fit: Various metrics were used to assess model fit. The RMSEA for the long-form exhibited an acceptable value of 0.06 (≤0.08 threshold indicates good model fit). The SRMSR was also acceptable at 0.075 (≤0.08 threshold indicates good fit). The Comparative Fit Index (CFI) value of 0.93 indicates substantial model improvement over a null model. The Tucker-Lewis Index (TLI) value was slightly below the good threshold at 0.914 (≥0.95 = good fit). The Chi-square value (M2) of 719.08, at *p* < 0.001 significance, suggested poorer model fit. However, in the overall context of the collective RMSEA, SRMSR, TLI, and CFI model performance statistics, the bi-factor model structure fit the data adequately.

### 3.4. 8 Item (Short-Form) Version Validation—Model Validation and Model Fit

Reliability: Empirical reliability of the short-form TfWQ for the older worker (50+ years) sample was 0.90, indicative of good to excellent internal consistency. The correlation between the model-based scores of the long- and short-forms was r = 0.97, reflecting high levels of agreement between both formats. Reliability of the short-form TfWQ in the older worker sample was high.

Model Validation: Table 6 shows the discrimination parameters and intercepts of the eight short-form items. Discrimination parameters for the items ranged from 1.51 to 3.94. Item 15 (“I am paid fairly for the job I do”) displayed the lowest discrimination parameter of 1.51, yet, remains above the classical test theory standard cutoffs for acceptability [78]. This is consistent with the conclusion that the discrimination parameter estimates do not threaten the short-form’s model acceptability. Loadings of IRT discrimination parameters are available in Appendix B (see Table A3). Overall, the short-form’s discrimination parameters were acceptable in the older worker sample.

Model Fit: Analysis for the short form was repeated, alternately using either item 24 (psychological safety) or item 25 (physical safety) as recommended by Peters and colleagues [14]. Discrimination parameters and model fit indices were comparable for both short-form models regardless of which item was used (psychological safety: empirical reliability = 0.90, M2[df 20] = 272.19, RMSEA = 0.15, SRMSR = 0.07, CFI = 0.94; physical safety: empirical reliability = 0.90, M2 [df 20] = 265.11, RMSEA = 0.15, SRMSR = 0.08, CFI = 0.93). RMSEA of 0.15 exceeded conventional cutoff values. The SRMSR was 0.07, indicating a reasonably good fit, while the CFI was high (0.94), demonstrating a close to strong fit.

The RMSEA of 0.15 was more than conventional cutoff values (~0.06 to 0.08), which warranted further investigation for potential model misfit [15]. Hence, a bootstrapping simulation was conducted (see Appendix C, Table A4 and Table A5). Results from the bootstrapping simulation showed that all observed values fell within the 2.5% and 97.5% confidence intervals of the simulated distributions, indicating no notable deviations between simulated and observed values. Contextualizing these findings, the analysis placed in the context of good SRMSR and CFI is indicative that the data fit the model well. Additionally, traditional cutoff values for fit indices such as RMSEA have been critiqued in the literature as arbitrary and may not universally apply across all modeling conditions or sample sizes [79]. Although RMSEA was elevated (0.15), bootstrap diagnostics showed no meaningful deviations and, together with favorable SRMSR and CFI, indicate acceptable overall model fit—also underscoring that rigid RMSEA cutoffs may not universally apply.

### 3.5. Construct Validation

To evaluate the external validity of the TfWQ, we conducted a series of correlational analyses between related validated measurement tools and TfWQ model-based scores for both the short-form (r_s_) and long-form (r_l_) TfWQ (see Table 7). TfWQ scores were expectedly positively correlated with a Cantril Ladder for life satisfaction (r_s_ = 0.35; r_l_ = 0.35) and best job (r_s_ = 0.58; r_l_ = 0.58), measures of job satisfaction (r_s_ = 0.67; r_l_ = 0.68), work engagement using the Well-BQ Job Engagement (r_s_ = 0.61; r_l_ = 0.62), and general wellbeing using the Flourishing Scale (r_s_ = 0.47; r_l_ = 0.50). There was an expected negative correlation with burnout (r_s_ = −0.58; r_l_ = −0.55), and turnover intention (r_s_ = −0.72; r_l_ = −0.72). Correlations using summative scoring did not differ significantly from correlations computed using TfWQ model-based scores (see Appendix D, Table A6). Construct validity using related but conceptually different measurement tools was established for both the long- and short-form TfWQs.

### 3.6. Thriving from Work Variation by Age Groups

Following satisfactory validation of the TfWQ among older workers, a comparative analysis was completed to identify if any substantive and meaningful differences existed on the TfWQ and the specific TfWQ items between the older (age 50+) and the younger (ages 20–49) worker samples. Scores for each thriving item were compared between older and younger workers using independent *t*-test comparisons. After controlling for multiple comparisons using the Benjamini-Hochberg procedure, 11 items remained statistically significant. Younger adults scored higher on opportunities for promotion. Older adults scored higher on psychological safety, physical safety, commuting, work-life balance, work-family, energy, resources, happiness, and life satisfaction (see Table 4). In addition, overall TfWQ scores did differ statistically between workers aged 20–49 compared to 50+, with older workers scoring, on average, higher thriving from work compared to younger workers (see Table 4). In summary, age differences did exist between younger and older workers with respect to overall thriving from work scores, and on nine items measuring TFW attributes.

## 4. Discussion

### 4.1. Validation Findings

This study demonstrates the valid utility of the TfWQ to measure work-related wellbeing among workers in the later stages (50+ years) of their work lives. We found that the long-form and short-form versions of the TfWQ (either with or without the omitted ‘hurt’ item) demonstrated adequate to good model fit within expected parameters, with bootstrapping simulations supporting the acceptable performance ranges where deviations occurred. However, as model fit substantially improved with the removal of the ‘hurt’ item, we recommend removing the ‘hurt’ item when using the long-form version unless the question is of specific interest to the research or practice use. All observed correlations with construct validation measures were in the expected direction and magnitude, providing strong evidence that the TfWQ captures a distinct but meaningfully related dimension of workplace wellbeing. The validation of the TfWQ in older workers aligns the thriving construct with established theories of aging regarding time horizons, lifespan development models, and occupational aging frameworks.

This validation study further confirmed a strong correlation between model-based and summed scores of the TfWQ instrument for both the long-form and short-forms. The summative scoring approach did not alter the direction or weaken the strength of aligned correlations between the TfWQ and the tested construct validation instruments. These findings support an implementer’s choice for using either model-based or summative scoring when using either the long-form or short-form version of the TfWQ that is appropriate for the applied context.

In classical test theory, when domains are conceptualized as explanatory factors contributing to a general factor, it is expected to find higher discrimination parameter values on the domains. The parameter indicates sensitivity to change in the latent construct. In this analysis of TfW among older workers, domains had moderately positive to slightly negative marginal discrimination parameters, which were lower than loadings on the TfW general factor. This alternative pattern is expected when the general factor captures most of the shared variance. This supports Peters et al’s., original conceptualization of Thriving from Work as a bi-factor model with items developed specifically for loading onto the general TfW factor first, and the remaining variance then attributed to the respective domains [14]. In this bi-factor conceptualization, the six domains are conceptually meaningful and should only be used as standalone measures of the domain if they demonstrate acceptable reliability parameters. Consistent with this application of the bifactor model proposed by Peters et al. [14], item factor loadings are expected to be larger on the general TfW factor than on the specific domain factors. This follows the pattern seen in the primary U.S. [14], Spanish [15], and German TfWQ validation studies [16], and was confirmed in our validation with an older worker US representative sample.

A single item, “I worry that I will get hurt at work,” was removed from the final model testing. The item had low factor loadings on both the general TfW and the specific corresponding wellbeing factor, suggesting little value or additional meaning for TfW measurement. The item also had a high ceiling effect after reverse scoring, with scores heavily skewed toward high levels of expected safety. It is possible that older workers in the US may be more likely to work in jobs that are in safe industries or conditions (e.g., office work) when compared to the full multi-generational workforce for which the item showed greater marginal utility. Thus, we recommend not including this item in the final long-form questionnaire when specifically studying workers aged 50+, resulting in a 29-item instrument. This item similarly had low utility for the Spanish [15] and German-language validation studies of the TfWQ [16]. Additional research may reevaluate the settings, conditions, or worker demographics for which safety concerns are most relevant to worker thriving in older workers, such as older workers in high-risk risk for injury jobs.

### 4.2. Age-Relevant Recommendations for Implementing the Thriving from Work Questionnaire in Practice

Our analysis confirms the reliable and valid use of the TfWQ instrument for older workers in practice.

#### 4.2.1. Demographic, Socio-Economic, and Occupational Variations

Although not the focus of our study, we did explore descriptive differences in TfW and the TfWQ items between younger (20–49) and older (50+) workers. We found differences across several attributes (i.e., items) of TfW and an overall increase in thriving across the lifespan. Another study by Lee et al., which focused on complete or overall well-being in U.S. workers, also found that differences in well-being and its dimensions increased with age [80]. Similar age-related findings have been found in prior studies, e.g., [81,82]. Our study, along with evidence from these prior studies, provides support for age-sensitive differences of wellbeing, either (a) being considered more important and/or (b) manifesting differently, across the lifespan. Our study did not focus on exploring other socio-demographic differences (such as race and ethnicity) in thriving from work (outside of age), but other studies, such as the aforementioned one conducted by Lee et al. [80], support that differences are likely to exist, identifying an important avenue for further study. Additionally, prior general wellbeing studies in employees have found demographic differences: for example, cultural across countries [83], ethnic within-country [84], sex [85,86], and income level [87], and occupation and skill level [88] differences. However, a review of empirical studies also yielded inconsistent results across demography, geography, and occupation, likely due to the variations in measurement and conceptualization of worker wellbeing and comparison groups [89]. This points to several key directions for future research and implications for practice into how cultural, socio-economic, demographic, and occupational contexts shape worker wellbeing across the lifespan.

Older and younger workers differed significantly on only a few items, which included all three items in the Work Life Integration subdomain being rated as higher for older workers, with no significant difference between workers aged 50–59 years and workers aged 60+ years, though these age brackets may be arbitrary from a life course perspective. This pattern of enhanced work-life balance may be expected when considering lifespan theories that suggest older workers are more likely to engage in work and career optimization strategies and job crafting, thus seeking and achieving greater integration between their job responsibilities and personal lives as they age [21,90]. Work-life integration might also be qualitatively different for older adults as responsibilities shift, or older adults may simply prioritize work-life balance and self-select into jobs or work roles that offer more of it [91]. Additionally, measurement considerations such as a positivity bias altering reporting or a surviving worker bias removing workers facing external demands (e.g., leaving work for caregiving or health challenges) may come into play [26]. The aligned demographic data from our sample of workers aged 60+ years reporting fewer hours worked per week may be impacted by any of these factors. Our findings emphasizing a unique deviation and positive tilt toward work-life integration among older workers highlight that these differences are practically different as well as significantly statistically different. This has the potential to inform organizational policy to prioritize thriving from work as a way of supporting older workers [92].

Other items showing improving trends by age indicated that workers who remained in the workforce at later ages had higher reports of psychological safety, more resources at work, and higher levels of work that shapes their happiness and contributes to their overall life satisfaction. The consistently higher ratings of psychological safety and meaningfulness among older workers align with predictions from social-emotional selectivity theory, that as time horizons narrow, older workers prioritize emotionally meaningful goals compared to their younger counterparts [19,93,94]. Given that labor force participation rate drops quickly past midlife [95], it remains uncertain whether the observed score differences primarily reflect intrinsic attributes of surviving workers, whether supportive organizational contexts contribute to thriving and retention, or if an interplay of factors is at play. Future interventions aimed at workforce retention may benefit from exploring these factors for extending work opportunities [21].

Relatively lower thriving scores on advancement opportunity among older workers match a national survey that found older workers received less on-the-job training and have fewer prospects for career advancement than younger workers [96]. Prior research shows no differences between younger and older workers on their satisfaction with opportunities for learning and development [97], which may reflect a developmental shift away from career-oriented striving. The score averages indicate this effect is not a feature of older workers having advanced to the top of their career field. Younger CEO’s, directors, and managers all reported higher levels of promotion opportunity compared to their position-matched older peers. Further demonstrating this point, older CEO’s, directors, and managers reported having more (not less) promotion opportunities than individual contributors who have no supervisory responsibility. Potential implications might suggest that older workers would benefit from additional career-building support. Organizations might build lateral advancement tracks [98], interventions to reduce age-bias in advancement or hiring [99] and ensure equitable access to job training with clarity around promotion pathways.

#### 4.2.2. Utility of Domain-Specific Measures

The three domains with strong reliabilities can offer domain-specific measurement and include (S5) job design and experience of work, (S3) work-life integration, and (S1) work-related emotional and psychological wellbeing. Job design is a recognized tactic for sustainable careers, and older workers scoring low in this area of thriving may benefit from work optimization or selective retraining that can build career longevity [21,90]. When older workers score low in work-life integration, organizations might consider proven strategies offering flexible work, phased-down hours, or tailored benefit packages for specific age groups [21], potentially supplementing childcare supports with a growing demand for eldercare policies [100]. The higher rates of psychological safety from the emotional and psychological wellbeing from work domain might recommend the practice of recruiting older workers as a resource for building positive relations between groups at work through mentoring or coaching support [21].

#### 4.2.3. Implications for Occupational Health Interventions and Policy 

The TfWQ has the potential to shape both the design and measurement of well-being across policy and practice. Measurement of TfW domains facilitates the identification of priority areas of need to direct the design of worker well-being interventions. Meta-analysis and systematic review evidence support the tailoring of workplace-based interventions for improving older workers’ health and well-being [101]. Moreover, the TfWQ also provides a reliable and valid measure to establish intervention effectiveness for improving older worker well-being that could provide a common comparison across studies or for benchmarking. Further, the need for age-sensitive interventions and policy has been supported internationally [102,103]. The NIOSH Productive Ageing framework and UN Decade of Healthy Ageing (2020–2030) recommend a life-course approach that integrates safety, health promotion, and multigenerational culture [104,105]. Regulatory guidance for employers also considers the need for lifespan approaches, with a 2024 Eurofound policy review identifying national incentives for flexible pensions, phased retirement, and digital up-skilling grants as policy levers that demonstrably delay labor-market exit [106]. Integrating TfWQ metrics into these frameworks could provide employers and policymakers a common measurement validated across the entire lifespan that is age-sensitive for monitoring progress, shaping policy, and targeting resources toward workers who could benefit most.

Further, when implementing the TfWQ into practice in a sample containing older workers with expected limited digital literacy or access, consider also using offline survey implementation methods such as paper-based mailed or community-based surveys or interviewer-administered surveys either by phone or in person (see also Section 4.5 Limitations).

### 4.3. Age-Relevant Recommendations for Using the Thriving from Work Questionnaire in Research

The validation of the TfWQ for older adults not only supports its use across age groups but also presents rich opportunities to analyze the existing data more deeply and draw practical inferences. Specific recommendations include examining TfW across different job and employment sectors to identify where age-related differences might be more pronounced to facilitate tailoring of workplace interventions to contexts where older versus younger workers’ thriving differs. Similarly, differences between work roles (e.g., manager versus frontline or administrative versus technical) could be explored in more depth to understand whether certain roles amplify or attenuate age disparities in thriving. Noting score differences between older and younger workers, future studies should monitor changes in thriving over time across the lifespan. Additionally, as age-related differences in thriving are identified, a lifespan perspective can guide the selection of interventions that can boost younger workers’ thriving levels to that of their older colleagues. With accurate measurement, attention to dips in thriving can identify the types of transitions and organizational shortcomings that warrant further research. Additionally, future research could explore qualitative differences in the manifestation of TfW in older workers compared to their younger counterparts. Qualitative and mixed methods research adds depth and meaning to the lived experience of older workers to explore how attributes within each of the TfW domains are perceived and experienced.

Though we assessed construct and convergent validity, we did not test predictive validity. Future studies should explore how TfWQ scores relate to meaningful outcomes for older workers, such as retirement timing, disability claims, long-term health, or continued labor force participation. This could help establish recommendations for age-sensitive workforce planning and interventions. Further, future analyses examining the interaction between age and other demographic and occupational variables and their influence on TfW would allow more in-depth examination of sociodemographic and occupational variations in how thriving from work is experienced across the lifespan.

As the TfWQ has been validated in multiple languages, cross-cultural validation studies could assess how different labor policies and social safety nets shape thriving for older workers across contexts. This may be particularly important, given the differences in policies and norms toward longer working lives and mandatory retirement across countries [107,108].

### 4.4. Sampling and Generalizability

Recruitment in this study was limited to English-speaking internet users; consequently, older adults who are digitally disconnected or have limited functional literacy were likely under sampled. This was demonstrated through our difficulty obtaining a sufficient representative sample for the 60+ age group for racial/ethnic minorities using the Prolific platform. Although the Prolific platform offers higher racial and educational diversity and built-in data-quality checks comparable to many crowdsourced research survey platforms, it remains an online, predominantly English-speaking non-probability panel. We did attempt to attain racial and ethnic diversity by oversampling minority racial and ethnic groups. Regardless, older adults with limited broadband access, very-low household incomes, limited English proficiency, or low digital literacy—many of whom are concentrated in manual, contingent, or informal occupations—are likely under-represented [109]. Because these groups often report lower job quality and wellbeing, the thriving scores observed here may represent upper-bound estimates for the broader ≥50-year U.S. workforce. Post-stratification weighting to U.S. age-sex-race benchmarks could reduce, but is still unlikely to eliminate this bias for internet-based recruitment and sampling methods [110]. Future studies and practical implementation of the TfWQ should consider employing multi-modal online and offline recruitment (e.g., telephone, interviewer-administered, mailed surveys, or community-based sampling approaches) to support surveying underrepresented populations and increase generalizability of the findings [111].

### 4.5. Limitations

This analysis of TfWQ long-form and short-form versions produced stable estimates within established parameters with demonstrated construct and convergent validity and reliability. Content validity was established in a previous study [13]. While this provides strong initial support for the validity of the TfWQ among older workers, several limitations warrant consideration. First, the lower factor loadings on domains and lower empirical reliability for some dimensions warrant interpretive caution and further study as described in Section 4.3.

Second, the study utilized a cross-sectional design, which limits the ability to assess change in thriving over time or to determine causal relationships between work conditions and wellbeing outcomes or sustainable extended work. Although not the intent of this study, it provides preliminary data to support future research pursuing longitudinal study designs to evaluate the stability and developmental trajectories of thriving throughout the lifespan, particularly across key life transitions that occur with aging, such as retirement, caregiving, spousal loss, or health-related work interruptions.

Third, although the sample was designed to be nationally U.S. representative in demographics with breadth across occupations and work roles, limitations in the sampling registry with Prolific—especially among workers aged 60+ years—resulted in underrepresentation of certain racial and ethnic groups. Future research could address this using different recruitment and survey deployment methods.

Fourth, the sample was limited by the necessity of validating worker thriving with currently working individuals. Given notable job precarity experienced by workers ages 50 and older [31,33], lower-thriving workers likely exit the workforce in higher numbers [26]. This would contribute to a selection bias toward higher average thriving scores for those who remain in the workforce. These factors limit generalizability and highlight the need for validation in more racially, culturally, and linguistically diverse populations, as well as across different industries and job types.

Finally, although we examined variability in income and occupation, we were only able to report these descriptively due to sample size limitations. Future research should explore under-represented occupational groups (such as more manual occupations and those with lower digital literacy versus professional service jobs) to capture wider cultural and occupational contexts.

## 5. Conclusions

We found that the long-form and short-form TfWQ are reliable and valid instruments for assessing work-oriented thriving among workers aged 50+ years and older. One item from the original questionnaire can be omitted from the long-form when administered to adults over age 50, resulting in a 29-item long-form instrument. The short-form remained unchanged. The short-form has added practical utility in employer-based or population-based surveys due to its strong reliability and validity in measuring the latent construct of thriving from work with only 8 items. We also found statistically significant differences between the scores of older and younger workers for the general factor TfW score. Compared to workers under age 50, older workers reported differences on nine factors, with the most pronounced differences with respect to perception of fewer promotion opportunities at work, but higher reports of work that produces happiness, life satisfaction, and higher experiences of psychological safety at work and work-life integration. The TfWQ instrument offers a valid and reliable tool for measuring work-related wellbeing among older workers. In addition, validation of the TFWQ in an older worker population provides support for the robust measurement of work-related wellbeing to support the need for age-inclusive wellbeing policies and workforce planning.

## Figures and Tables

**Table 1:** Thriving from Work (TfW) Conceptualization and Description.

TfW Domain	Domain Description	Attributes of the Domain	Example Item
Emotional & Psychological Wellbeing from Work (S1)	State of positive emotional and psychological wellbeing because of work	Meaning and purpose Growth and development Job satisfaction Happiness Love the job you have Contribution to life satisfaction	I love my job.
Social Wellbeing from Work (S2)	Key relational aspects at work supporting worker wellbeing	Supported Valued Belonging Respect Fair treatment Voice Recognition	I am treated fairly at work.
Work Life Integration (S3)	Interface between work and life outside of work and how these integrate to support wellbeing	Work-life balance Work-family balance Commuting	I can achieve a healthy balance between my work and my life outside of work
Basic needs for Thriving from Work (S4)	The basic needs necessary to provide a foundation for worker wellbeing	Fair pay Job security Health and wellbeing benefits Promotion opportunity	I am paid fairly for the job I do.
Job Design and Experience of work (S5)	Critical elements of job design and working conditions support worker wellbeing	Autonomy Schedule control Reasonable job demands Adequate resources to do the job well Work intensity	I am happy with how much input I have in decisions that affect my work.
Physical and Mental Health and Wellbeing from Work (S6)	Work’s contribution to physical and mental health and wellbeing	Physical safety Psychological safety Fear of injury Job stress Energy	I can easily manage the demands of my job.

**Table 2:** Worker characteristics of all age group samples.

Characteristics	Age Group (20–49) N = 372	Age Group (50–59) N = 352	Age Group (60+) N = 265
TfW Total Score mean (SD)	122.18 (27.46)	124.31 (27.94)	130.51 (25.86)
Age mean (SD)	34.84 (8.24)	53.83 (2.90)	64.48 (4.60)
Race/Ethnicity (n (%))			
White	163 (43.81%)	174 (49.43%)	161 (60.75%)
Black/African American	83 (22.31%)	77 (21.88%)	58 (21.89%)
Asian	36 (9.68%)	24 (6.82%)	5 (1.89%)
Native American/Alaskan Native	12 (3.23%)	10 (2.84%)	4 (1.51%)
Hispanic/Latino	59 (15.86%)	51 (14.49%)	17 (6.42%)
Other (Mixed)	19 (5.11%)	16 (4.55%)	20 (7.55%)
Not reported	0	0	0
Sex n (%)			
Male	136 (36.55%)	155 (44.03%)	98 (36.98%)
Female	146 (39.25%)	195 (55.40%)	163 (61.51%)
Other/Rather not say	11 (0.3.96%)	2 (0.56%)	4 (1.5%)
Not reported	79 (21.24%)	0	0
Highest level of education n (%)			
No formal qualifications	2 (0.54%)	0 (0.00%)	0 (0.00%)
High School	66 (17.74%)	82 (23.30%)	66 (24.91%)
Technical/community college	47 (12.63%)	70 (19.89%)	67 (25.28%)
Undergraduate degree (e.g., Bachelor)	166 (44.62%)	143 (40.63%)	81 (30.57%)
Graduate degree (e.g., Masters)	80 (21.50%)	48 (13.64%)	42 (15.85%)
Doctorate degree (e.g., PhD)	10 (2.69%)	9 (2.56%)	9 (3.40%)
Not reported	1 (0.54%)	0 (0.00%)	0 (0.00%)
Number of employers last month n (%)			
0	8 (2.15%)	2 (0.57%)	4 (1.51%)
1	287 (77.15%)	280 (79.55%)	205 (77.36%)
2	24 (6.45%)	32 (9.09%)	17 (6.42%)
3 or more	53 (14.25%)	38 (10.80%)	39 (14.71%)
Not reported	0	0	0
Worked < 31 h per week n (%)	87 (23.39%)	70 (19.89%)	101 (38.11%)
Not reported	0	0	0
Annual household USD Income n (%)			
$0–$19,999	26 (6.99%)	27 (7.68%)	32 (12.07%)
$20,000–$49,999	57 (15.32%)	81 (23.01%)	70 (26.41%)
$50,000–$79,999	76 (20.43%)	76 (21.59%)	74 (27.93%)
$80,000–$149,999	97 (26.07%)	105 (29.83%)	64 (24.15%)
More than $150,000	37 (9.95%)	63 (17.90%)	25 (9.43%)
Not reported	79 (21.24%)	0	0
Work Role (n/%)			
C-suite/Executive	8 (2.15%)	18 (5.11%)	10 (3.77%)
VP/Director	19 (5.11%)	25 (7.1%)	17 (6.42%)
Manager/Senior Manager	109 (29.30%)	125 (35.51%)	83 (31.32%)
Employees/individual contributor	226 (60.75%)	169 (48.01%)	130 (49.06%)
Owners/Partners	10 (2.69%)	15 (4.26%)	25 (9.43%)
Not reported	0	0	0
Job Sector (n/%)			
Management, Professional & Related	239 (64.2%)	214 (60.8%)	148 (55.85%)
Service Occupations	33 (8.9%)	34 (9.66%)	38 (14.34%)
Sales & Office	60 (16.1%)	75 (21.31%)	58 (21.88%)
National Resources, Construction, & Maintenance	15 (4%)	12 (3.41%)	8 (3.02%)
Production, Transportation & Material Moving	24 (6.5%)	17 (4.83%)	13 (4.91%)
Not reported	1 (0.3%)	0	0

**Table 3:** Thriving from Work Questionnaire item distribution (% of total responses for each item) for workers aged 50 years and older.

No.	Item	Never	Rarely	Sometimes	Usually	Almost Always	Always	NA
**Work-related Emotional & Psychological Wellbeing (S1)**								
1	I love my job	5.42	12.06	17.66	21.33	25.17	18.36	0.65
2	My work adds meaning to my life.	6.10	13.24	19.69	20.56	23.52	23.52	0.49
3	My job allows me to achieve my full potential.	8.92	13.46	21.33	19.76	22.03	14.51	0.82
4	The kind of work I do makes me happy.	4.36	8.89	17.07	23.17	26.48	20.03	0.16
5	I am satisfied with my job.	3.83	8.01	17.07	20.73	29.62	20.73	0.33
6	My work adds to my overall life satisfaction.	5.06	12.04	18.32	21.99	26.35	16.23	0.65
**Social Wellbeing from Work (S2)**								
7	I am treated fairly at work.	1.57	4.88	11.15	19.69	29.09	33.62	0.81
8	I feel supported by the people I work with.	2.10	6.47	15.21	19.76	27.97	28.50	1.95
9	I feel valued by the people I work with.	2.79	7.68	15.36	21.29	24.78	28.10	1.78
10	I am treated with respect at work.	1.39	4.18	10.98	17.42	29.27	36.76	0.97
11	At work, I feel like I belong.	4.36	7.49	13.07	22.13	23.17	29.79	0.97
**Work-life Integration (S3)**								
12	I can achieve a healthy balance between my work and my life outside of work.	1.39	4.70	10.10	21.43	31.36	31.01	0.16
13	I can easily manage my job as well as attend to my needs and the needs of my family.	1.05	2.61	11.15	20.73	31.36	33.10	0.00
14	I feel safe getting to and from work.	0.54	0.54	3.08	15.94	25.36	54.53	7.29
**Basic Needs for Thriving (S4)**								
15	I am paid fairly for the job I do.	6.27	9.41	14.11	24.04	23.52	22.65	0.16
16	I am satisfied with the amount of paid leave.	7.75	7.56	15.12	18.34	27.60	23.63	9.73
17	I feel my job is secure.	5.75	5.92	15.16	25.78	23.17	24.22	0.33
18	I have good opportunities for promotion.	15.49	25.93	19.59	15.49	14.18	9.33	8.27
**Job Design & Experience of Work (S5)**								
19	I am happy with how much input I have in decisions that affect my work.	6.47	9.09	19.76	25.00	24.83	14.86	1.46
20	I can easily manage the demands of my job.	0.87	2.61	12.02	21.25	33.10	30.14	0.16
21	I have adequate control over the pace of my work.	4.01	8.55	13.44	27.05	27.75	19.20	0.33
22	I am happy with how much control I have over my work schedule.	6.14	7.89	17.19	22.63	23.16	22.98	0.81
23	I have access to the resources I need to do my job well.	0.87	2.97	13.29	21.68	30.59	30.59	0.49
**Physical and Mental Health and Wellbeing from Work (S6)**								
24	I feel psychologically safe at work.	1.40	2.45	10.49	19.58	23.78	42.31	1.46
25	I feel physically safe at work.	0.70	1.05	4.38	14.19	25.57	54.12	3.08
26	I feel excessive levels of stress from my work. *	3.67	6.12	9.79	32.69	33.22	14.51	0.49
27	After I leave work, I have enough energy to do the things I want or need to do.	2.44	8.90	21.12	27.92	24.43	15.18	0.81
28	I worry that I will get hurt at work. *	1.69	1.31	2.81	10.30	25.09	58.80	10.37
29	I can voice concerns or make suggestions at work without getting into trouble. **	2.11	7.02	14.21	21.58	22.63	32.46	1.46
30	I receive recognition at work for my accomplishments. **	6.11	12.39	24.61	19.90	20.59	16.40	0.81

Sample size for older worker sample (N) = 617. Note, there were no missing items for any TfWQ items. NA = “Not Applicable” response, treated as missing for analysis. * Items 26 and 28 are reverse-coded for analysis. ** Items 29 and 30 load only onto the general factor.

**Table 4:** Descriptive statistics for Thriving from Work Questionnaire items and comparison of older and younger worker responses (n = valid responses included in analysis).

Item		Mean Age 50+	SD Age 50+	Mean Age 20–49	SD Age 20–49	Skew Age 50+	Kurtosis Age 50+	Adj. *p*-Value **
**Work-related Emotional & Psychological Wellbeing (S1)**								
1	I love my job.	4.04	1.46	3.88	1.49	−0.36	−0.84	0.20
2	My work adds meaning to my life.	3.93	1.48	3.74	1.49	−0.26	−0.93	0.11
3	My job allows me to achieve my full potential.	3.76	1.52	3.64	1.48	−0.19	−0.98	0.30
4	The kind of work I do makes me happy.	4.19	1.40	3.91	1.42	−0.47	−0.60	**0.02**
5	I am satisfied with my job.	4.26	1.38	4.06	1.40	−0.54	−0.53	0.06
6	My work adds to my overall life satisfaction.	4.01	1.43	3.78	1.46	−0.35	−0.80	0.05
**Social Wellbeing from Work (S2)**								
7	I am treated fairly at work.	4.71	1.26	4.61	1.16	−0.83	−0.01	0.28
8	I feel supported by the people I work with.	4.51	1.33	4.39	1.22	−0.63	−0.45	0.24
9	I feel valued by the people I work with.	4.42	1.38	4.32	1.34	−0.57	−0.58	0.35
10	I am treated with respect at work.	4.79	1.24	4.72	1.17	−0.91	0.13	0.40
11	At work, I feel like I belong.	4.42	1.44	4.22	1.29	−0.65	−0.47	0.07
**Work Life Integration (S3)**								
12	I can achieve a healthy balance between my work and my life outside of work.	4.70	1.22	4.48	1.23	−0.83	0.12	**0.03**
13	I can easily manage my job as well as attend to my needs and the needs of my family.	4.78	1.16	4.51	1.25	−0.81	0.13	**0.01**
14	I feel safe getting to and from work.	5.29	0.94	5.04	1.13	−1.37	1.98	**0.01**
**Basic Needs for Thriving (S4)**								
15	I am paid fairly for the job I do.	4.17	1.48	4.02	1.51	−0.51	−0.65	0.28
16	I am satisfied with the amount of paid leave I can take to care for myself or family members.	4.21	1.53	4.11	1.57	−0.61	−0.62	0.06
17	I feel my job is secure.	4.27	1.43	4.18	1.41	−0.58	−0.39	0.39
18	I have good opportunities for promotion.	3.15	1.56	3.48	1.53	0.32	−1.01	**0.01**
**Job Design & Experience of Work (S5)**								
19	I am happy with how much input I have in decisions that affect my work.	3.97	1.41	4.40	1.29	−0.38	−0.63	0.97
20	I can easily manage the demands of my job.	4.74	1.14	4.56	1.15	−0.73	0.00	0.06
21	I have adequate control over the pace of my work.	4.24	1.35	4.18	1.25	−0.56	−0.37	0.57
22	I am happy with how much control I have over my work schedule.	4.18	1.47	4.04	1.41	−0.49	−0.65	0.22
23	I have access to the resources I need to do my job well.	4.70	1.17	4.49	1.18	−0.67	−0.22	**0.03**
**Physical and Mental Health and Wellbeing from Work (S6)**								
24	I feel psychologically safe at work.	4.89	1.21	4.55	1.26	−0.94	0.23	<0.001
25	I feel physically safe at work.	5.25	1.00	5.02	1.12	−1.45	2.08	**0.01**
26	I feel excessive levels of stress from my work. *	4.29	1.23	4.13	1.31	−0.77	0.36	0.11
27	After I leave work, I have enough energy to do the things I want or need to do.	4.09	1.28	3.88	1.34	−0.26	−0.60	0.05
28	I worry that I will get hurt at work. *	5.32	1.04	5.11	1.28	−1.98	4.36	**0.03**
29	I can voice concerns or make suggestions at work without getting into trouble.	4.53	1.36	3.97	1.28	−0.61	−0.55	0.21
30	I receive recognition at work for my accomplishments.	3.86	1.46	3.94	1.36	−0.14	−0.92	0.43
	**Long-form TfWQ (Summed)**	126.39	27.53	122.18	27.46	−0.46	2.46	**0.02**

Sample size (N) = 989. * Items 26 and 28 are reverse-scored. Significant values < 0.05 are in bold. ** *t*-test comparisons between younger and older workers (See Section 3.6).

**Table 5:** Thriving from Work Questionnaire Long-form marginal discrimination parameters for workers aged 50+ years for the general latent construct of thriving from work and for each domain (S1–S6).

	Item	General	S1	S2	S3	S4	S5	S6
**Work-related Emotional & Psychological Wellbeing (S1)**								
1	I love my job	1.6974	1.3228					
2	My work adds meaning to my life.	1.2260	1.8284					
3	My job allows me to achieve my full potential.	1.6867	1.0444					
4	The kind of work I do makes me happy.	1.5082	1.4552					
5	I am satisfied with my job.	2.0865	1.0061					
6	My work adds to my overall life satisfaction.	1.3849	1.6287					
**Social Wellbeing from Work (S2)**								
7	I am treated fairly at work.	3.6294		0.3935				
8	I feel supported by the people I work with.	2.4598		0.9537				
9	I feel valued by the people I work with.	2.3845		0.9926				
10	I am treated with respect at work.	3.1055		0.4590				
11	At work, I feel like I belong.	2.6205		0.6349				
**Work Life Integration (S3)**								
12	I can achieve a healthy balance between my work and my life outside of work.	1.3899			1.7001			
13	I can easily manage my job as well as attend to my needs and the needs of my family.	1.3007			1.9571			
14	I feel safe getting to and from work.	0.9401			0.6308			
**Basic Needs for Thriving (S4)**								
15	I am paid fairly for the job I do.	1.4755				0.9178		
16	I am satisfied with the amount of paid leave I can take to care for myself or family members.	1.2381				0.9631		
17	I feel my job is secure.	1.5537				0.3406		
18	I have good opportunities for promotion.	1.3169				0.5357		
**Job Design & Experience of Work (S5)**								
19	I am happy with how much input I have in decisions that affect my work.	2.2622					0.3524	
20	I can easily manage the demands of my job.	1.4176					0.6482	
21	I have adequate control over the pace of my work.	1.5824					1.2846	
22	I am happy with how much control I have over my work schedule.	1.3744					1.1892	
23	I have access to the resources I need to do my job well.	1.7867					0.1096	
**Physical and Mental Health and Wellbeing from Work (S6)**								
24	I feel psychologically safe at work.	1.7353						0.9945
25	I feel physically safe at work.	1.0746						1.1410
26	I feel excessive levels of stress from my work.	0.9812						0.3735
27	After I leave work, I have enough energy to do the things I want or need to do.	1.2806						0.5747
29	I can voice concerns or make suggestions at work without getting into trouble.	2.1853						
30	I receive recognition at work for my accomplishments.	2.0500						
	*Empirical reliability*	0.9385	0.8378	0.5795	0.7429	0.4955	0.6522	0.5170

Sample size (N) = 574, M2_(df = 234)_ = 719.08, *p* < 0.001, RMSEA = 0.06, SRMSR = 0.08, CFI = 0.93. Items 29 and 30 are not associated with a specific domain and only load to the general TfW factor. Item 28 was not included. ‘Not Applicable’ responses were treated as missing for this analysis. The MHRM estimator was used to calculate the above marginal discrimination parameters.

**Table 6:** Thriving from Work Questionnaire Short-form discrimination parameters and category intercepts for workers ages 50 years and over.

	Long-Form Items	Discrimination Parameter	Category Intercepts				
			1	2	3	4	5
1	I love my job.	1.80	4.10	2.34	0.91	−0.43	−2.25
7	I am treated fairly at work.	3.94	9.18	6.62	4.10	1.32	−1.80
12	I can achieve a healthy balance between my work and life outside of work.	1.60	5.39	3.66	2.31	0.71	−1.16
15	I am paid fairly for the job I do.	1.51	3.54	2.31	1.25	−0.17	−1.68
19	I am happy with how much input I have in decisions that affect my work.	2.45	4.60	3.14	1.18	−0.75	−3.13
20	I can easily manage the demands of my job.	1.61	5.92	4.32	2.33	0.73	−1.23
24	I feel psychologically safe at work.	2.07	6.09	4.81	2.85	1.04	−0.56
29	I can voice my concerns or make suggests at work without getting into trouble.	2.41	6.12	4.02	2.18	0.36	−1.31
	*Empirical reliability*	0.90					

Model fit: M2_(df = 20)_ = 272.19; *p* < 0.001; RMSEA = 0.148; SRMSR = 0.07; CFI = 0.94. Sample size = 574.

**Table 7:** Pearson correlation to assess convergent validity for Thriving from Work Questionnaire model-based scores for workers ages 50+ years.

	Variables	1	2	3	4	5	6	7	8	9
1	TfW (long)	1.00								
2	TfW (short)	0.97	1.00							
3	Cantril Ladder for thriving	0.35	0.35	1.00						
4	Cantril Ladder for best job	0.58	0.58	0.62	1.00					
5	Job satisfaction	0.68	0.67	0.41	0.63	1.00				
6	Well-BQ job engagement	0.62	0.61	0.35	0.56	0.64	1.00			
7	Flourishing	0.50	0.47	0.47	0.45	0.42	0.51	1.00		
8	Burnout	−0.55	−0.58	−0.24	−0.41	−0.56	−0.52	−0.45	1.00	
9	Turnover intention	−0.71	−0.72	−0.33	−0.59	−0.73	−0.62	−0.41	0.57	1.00

Sample size N = 574.

## Data Availability

Data are available by request to the corresponding author.

## Abbreviations

The following abbreviations are used in this manuscript:

CFA	Confirmatory factor analysis
CFI	Comparative fit Index
CI	Confidence Interval
COPSOQ	Copenhagen Psychosocial Questionnaire
ESEM	Exploratory structural equation modeling
IRT	Item Response Theory
NIOSH	National Institute of Occupational Safety and Health
RMSEA	Root mean squared error of approximation
SRMSR	Standardized Root Mean-Square Residual
TfWQ	Thriving from Work Questionnaire
TfW	Thriving from work
TLI	Tucker-Lewis Index
US	United States

## Appendix A. Polychoric Intercorrelations

**Table A1:** Polychoric Correlations for all 30 original long-form items.

	1	2	3	4	5	6	7	8	9	10	11	12	13	14	15	16	17	18	19	20	21	22	23	24	25	26	27	28	29	30
**1**	1.00																													
**2**	0.83																													
**3**	0.83	0.79																												
**4**	0.87	0.86	0.80																											
**5**	0.87	0.79	0.82	0.84																										
**6**	0.85	0.89	0.79	0.84	0.82																									
**7**	0.62	0.55	0.64	0.60	0.70	0.58																								
**8**	0.63	0.57	0.65	0.61	0.67	0.59	0.84																							
**9**	0.64	0.60	0.67	0.60	0.69	0.62	0.83	0.92																						
**10**	0.61	0.50	0.62	0.57	0.67	0.54	0.91	0.83	0.83																					
**11**	0.69	0.63	0.68	0.69	0.73	0.64	0.83	0.85	0.86	0.82																				
**12**	0.50	0.46	0.43	0.50	0.59	0.47	0.56	0.52	0.52	0.54	0.55																			
**13**	0.48	0.46	0.42	0.50	0.57	0.47	0.52	0.51	0.51	0.51	0.52	0.90																		
**14**	0.29	0.27	0.26	0.34	0.34	0.29	0.47	0.42	0.37	0.51	0.45	0.54	0.56																	
**15**	0.51	0.45	0.59	0.48	0.60	0.49	0.58	0.55	0.54	0.54	0.53	0.44	0.41	0.30																
**16**	0.48	0.43	0.53	0.44	0.53	0.44	0.52	0.53	0.53	0.50	0.52	0.45	0.43	0.28	0.63															
**17**	0.51	0.47	0.56	0.46	0.59	0.53	0.63	0.64	0.63	0.58	0.61	0.48	0.46	0.34	0.51	0.52														
**18**	0.57	0.50	0.64	0.51	0.58	0.49	0.54	0.53	0.56	0.51	0.52	0.32	0.28	0.17	0.56	0.47	0.51													
**19**	0.67	0.56	0.68	0.61	0.69	0.59	0.72	0.67	0.66	0.68	0.69	0.44	0.44	0.31	0.56	0.52	0.54	0.59												
**20**	0.48	0.44	0.38	0.50	0.55	0.47	0.56	0.48	0.47	0.54	0.50	0.68	0.69	0.49	0.35	0.36	0.48	0.28	0.48											
**21**	0.53	0.43	0.51	0.52	0.58	0.49	0.59	0.54	0.53	0.57	0.55	0.57	0.57	0.39	0.45	0.45	0.45	0.42	0.71	0.63										
**22**	0.51	0.44	0.44	0.52	0.57	0.47	0.55	0.50	0.50	0.51	0.52	0.61	0.60	0.44	0.42	0.44	0.39	0.34	0.59	0.64	0.74									
**23**	0.56	0.48	0.53	0.55	0.60	0.50	0.67	0.64	0.58	0.66	0.63	0.58	0.57	0.46	0.48	0.41	0.49	0.40	0.58	0.62	0.52	0.50								
**24**	0.48	0.44	0.46	0.51	0.57	0.46	0.69	0.64	0.59	0.68	0.64	0.58	0.54	0.55	0.45	0.39	0.52	0.33	0.54	0.60	0.52	0.50	0.60							
**25**	0.31	0.31	0.31	0.36	0.40	0.32	0.51	0.46	0.44	0.51	0.45	0.49	0.48	0.67	0.38	0.37	0.38	0.21	0.38	0.51	0.44	0.46	0.47	0.68						
**26**	0.38	0.32	0.28	0.35	0.41	0.34	0.43	0.32	0.36	0.41	0.39	0.56	0.47	0.28	0.32	0.30	0.29	0.24	0.37	0.50	0.42	0.39	0.34	0.46	0.28					
**27**	0.46	0.40	0.43	0.45	0.54	0.41	0.49	0.40	0.41	0.48	0.48	0.63	0.59	0.36	0.44	0.40	0.35	0.36	0.41	0.56	0.49	0.45	0.47	0.57	0.47	0.49				
**28**	0.07	0.08	0.06	0.11	0.13	0.07	0.27	0.18	0.16	0.27	0.23	0.33	0.35	0.44	0.19	0.22	0.12	0.08	0.19	0.31	0.25	0.30	0.34	0.36	0.60	0.37	0.26			
**29**	0.49	0.39	0.52	0.50	0.54	0.41	0.76	0.70	0.67	0.72	0.70	0.48	0.43	0.40	0.46	0.46	0.52	0.48	0.71	0.45	0.60	0.51	0.55	0.61	0.47	0.33	0.44	0.23		
**30**	0.56	0.50	0.60	0.50	0.62	0.49	0.74	0.73	0.78	0.73	0.71	0.39	0.38	0.29	0.54	0.49	0.54	0.66	0.68	0.36	0.49	0.44	0.53	0.48	0.37	0.28	0.43	0.10	0.67	1.00

Polychoric correlations were estimated from ordinal items. All variables were measured on a 7-point Likert scale, including “Not Applicable” coded as ‘0’. Item 26 displayed the lowest intercorrelations and was removed from the ‘Thriving from Older Workers’ Questionnaire’.

## Appendix B. Mapping Discrimination Parameters as Factor Loadings

Table A2 and Table A3 of Appendix B present the factor loadings derived from item response theory (IRT) models using the summary () function from the ‘mirt’ package. This function allows us to extract the factor loadings from the bi-factor model, which are comparable to the factor loadings that you would expect from a Confirmatory Factor Analysis Model. This method is required as bi-factor modelling is not possible using traditional CFA methods. Table A2 shows the loadings from a bifactor model applied to the long-form, where each item loads on both a general factor and a specific domain factor. In contrast, Table A3 displays the loadings from a unidimensional model fitted to the short form, where items load solely on a single general factor, with no specific factors modeled. Although these loadings originate from IRT frameworks, they are conceptually analogous to those found in confirmatory factor analysis (CFA) and can be interpreted similarly. However, it is important to note that loadings derived independently from CFA may differ due to differences in modeling approaches. The purpose of these tables is to assist interpretation, particularly for readers less familiar with IRT-based analyses.

**Table A2:** Factor loadings for the long-form Thriving from Work Questionnaire.

	Item	General Thriving from Work	1	2	3	4	5	6
**(S1) Work-related Emotional & Psychological Well-being**								
1	I love my job	0.71	0.61					
2	My work adds meaning to my life.	0.58	0.73					
3	My job allows me to achieve my full potential.	0.70	0.52					
4	The kind of work I do makes me happy.	0.66	0.65					
5	I am satisfied with my job.	0.78	0.51					
6	My work adds to my overall life satisfaction.	0.63	0.69					
**(S2) Social Well-being from Work**								
7	I am treated fairly at work.	0.91		0.23				
8	I feel supported by the people I work with.	0.82		0.49				
9	I feel valued by the people I work with.	0.81		0.50				
10	I am treated with respect at work.	0.88		0.26				
11	At work, I feel like I belong.	0.84		0.35				
**(S3) Work-life Integration**								
12	I can achieve a healthy balance between my work and my life outside of work.	0.63			0.71			
13	I can easily manage my job as well as attend to my needs and the needs of my family.	0.61			0.76			
14	I feel safe getting to and from work.	0.48			0.35			
**(S4) Basic Needs for Thriving**								
15	I am paid fairly for the job I do.	0.66				0.48		
16	I am satisfied with the amount of paid leave I can take to care for myself or family members.	0.59				0.49		
17	I feel my job is secure.	0.67				0.20		
18	I have good opportunities for promotion.	0.61				0.30		
**(S5) Job Design & Experience of Work**								
19	I am happy with how much input I have in decisions that affect my work.	0.80					0.20	
20	I can easily manage the demands of my job.	0.64					0.36	
21	I have adequate control over the pace of my work.	0.68					0.60	
22	I am happy with how much control I have over my work schedule.	0.63					0.57	
23	I have access to the resources I need to do my job well.	0.72					0.06	
**(S6) Health, Physical, and Mental Well-being from Work**								
24	I feel psychologically safe at work.	0.71						0.51
25	I feel physically safe at work.	0.53						0.56
26	I feel excessive levels of stress from my work. **	0.50						0.21
27	After I leave work, I have enough energy to do the things I want or need to do.	0.60						0.32
29	I can voice concerns or make suggestions at work without getting into trouble.	0.79						
30	I receive recognition at work for my accomplishments.	0.77						

Sample Size (N) = 574. ** Item 28 was removed.

**Table A3:** Factor loadings for Thriving from Work Questionnaire Short Version.

Long-Form Item No.	Item	Loadings
1	I love my job.	1.80
7	I am treated fairly at work.	3.94
12	I can achieve a healthy balance between my work and life outside of work.	1.60
15	I am paid fairly for the job I do.	1.51
19	I am happy with how much input I have in decisions that affect my work.	2.45
20	I can easily manage the demands of my job.	1.61
24	I feel psychologically safe at work.	2.07
29	I can voice concerns or make suggestions at work without getting into trouble.	2.41

Sample Size (N) = 574.

## Appendix C. Parametric Bootstrapping Simulations for the TfWQ Short Version

To investigate our model fit, we also conducted parametric bootstrapping for each item of the TfW short form. This approach involves simulating data under the model’s assumptions for all short-form items. By running these simulations, our goal was to examine the relationship between the simulated data and the observed data. If the simulated data closely align with the observed data, this indicates that the model’s assumptions are appropriate.

The distribution of the response categories for each item in the bootstrapped samples should closely match the statistics of the observed data if the model is a good overall fit. In other words, we considered the model to demonstrate a good fit if the observed values fell within the simulated confidence intervals.

To identify potential sources of model misfit, we used the ‘item fit’ function from the ‘mirt package’ to calculate item-level fit statistics for all items in the short form of TfW. The chi-square statistic and corresponding *p*-values indicated that Item 20 could potentially contribute to model misfit, due to its significant *p*-value (*p* < 0.001, indicating significant deviation from the expected fit) despite a favorable RMSEA (0.03).

**Table A4:** Item fit TfWQ Short-form Version.

	Item	χ2	df	RMSEA	*p*
1	I love my job.	54.13	70	0.00	0.92
7	I am treated fairly at work.	43.70	40	0.01	0.32
12	I can achieve a healthy balance between my work and my life outside of work.	65.01	63	0.01	0.41
15	I am paid fairly for the job I do.	100.69	84	0.02	0.10
19	I am happy with how much input I have in decisions that affect my work.	76.33	59	0.02	0.06
20	I can easily manage the demands of my job.	92.46	58	0.03	0.00
24	I feel psychologically safe at work.	66.51	53	0.02	0.10
29	I can voice concerns or make suggestions at work without getting into trouble.	70.65	57	0.02	0.11

Sample Size (N) = 574.

However, the results from the parametric bootstrapping simulations indicated no notable deviations between the simulated and observed values. All observed values fell within the lower (2.5%) and upper (97.5%) bounds of the simulated confidence intervals. In the context of model fit, it is important to acknowledge that traditional cutoff values for fit indices—such as the RMSEA—have been critiqued in the literature for their somewhat arbitrary nature. Although our model yielded slightly elevated fit indices (e.g., RMSEA = 0.15), the results from the parametric bootstrapping simulations support the robustness and appropriateness of the model.

**Table A5:** Bootstrapped Simulation results for TfWQ Short-form Version.

			Response Category					
	Item	CI	1	2	3	4	5	6
1	I love my job.	2.5%	20	56	88	102	121	89
		97.5%	41	85	123	141	161	123
		observed	31	69	101	122	144	105
7	I am treated fairly at work.	2.5%	5	18	48	100	140	172
		97.5%	16	37	76	139	182	216
		observed	9	28	64	113	167	193
12	I can achieve a healthy balance between my work and my life outside of work.	2.5%	3	18	44	104	157	157
		97.5%	14	39	72	145	199	199
		observed	8	27	58	123	180	178
15	I am paid fairly for the job I do.	2.5%	26	40	61	169	118	113
		97.5%	50	67	93	156	158	151
		observed	36	54	81	138	135	130
19	I am happy with how much input I have in decisions that affect my work.	2.5%	26	35	95	123	125	70
		97.5%	49.03	62	133	166	166	103
		observed	37	52	113	143	142	85
20	I can easily manage the demands of my job.	2.5%	1	9	56	104	164	152
		97.5%	10	24	88	143	207	194
		observed	5	15	69	122	190	173
24	I feel psychologically safe at work.	2.5%	3	8	47	97	115	219
		97.5%	14	23	77	135	153	262
		observed	8	14	60	112	136	242
29	I can voice concerns or make suggestions at work without getting into trouble.	2.5%	7	28	66	107	107	168
		97.5%	20	52	98	144	146	212
		observed	12	40	81	123	129	185

Sample Size (N) = 574; CI = Confidence Interval.

## Appendix D. Correlation Between Sum Scores of the ‘Thriving’ Items from Both the Long-Form and Short-Form Versions with Validation Constructs

**Table A6:** Correlation between the sum scores of the Thriving from Work Questionnaire long-form and short-form versions and validation constructs.

Variables	TfWQ (Short-Form)	TfWQ (Long-Form)
TfWQ (long)	0.97	1.00
TfWQ (short)	1.00	0.97
Overall Life Thriving (Gallup Cantril Ladder)	0.38	0.41
Best job (Gallup Cantril Ladder)	0.61	0.63
Job satisfaction	0.71	0.74
Work engagement (Well-BQ Job Engagement)	0.64	0.70
Overall wellbeing (Flourishing)	0.50	0.55
Burnout	−0.58	−0.61
Turnover Intention	−0.72	−0.75

Sample Size (N) = 574, *p* < 0.001 (highly significant).
